# Gender Difference in Cisplatin-Induced Nephrotoxicity in a Rat Model: Greater Intensity of Damage in Male Than Female

**DOI:** 10.5812/numonthly.10128

**Published:** 2013-06-25

**Authors:** Mehdi Nematbakhsh, Shadi Ebrahimian, Mona Tooyserkani, Fatemeh Eshraghi-Jazi, Ardeshir Talebi, Farzaneh Ashrafi

**Affiliations:** 1 Water and Electrolytes Research Center, Isfahan University of Medical Sciences, Isfahan, IR Iran; 2 Department of Physiology, Isfahan University of Medical Sciences, Isfahan, IR Iran; 3 Kidney Diseases Research Center, Isfahan University of Medical Sciences, Isfahan, IR Iran; 4 Department of Clinical Pathology, Isfahan University of Medical Sciences, Isfahan, IR Iran; 5 Department of Internal Medicine, Isfahan University of Medical Sciences, Isfahan, IR Iran

**Keywords:** Cisplatin, Gender, Rats, Renal Insufficiency

## Abstract

**Background:**

Nephrotoxicity and hepatotoxicity are side effects of Cisplatin (CP) therapy.

**Objectives:**

We investigated the role of gender in CP-induced nephrotoxicity and hepatotoxicity.

**Materials and Methods:**

Low dose of CP (1 mg/kg/day; ip) was administered daily to male and female Wistar rats for 15 consecutive days. Serum creatinine (Cr), blood urea nitrogen (BUN), malondialdehyde (MDA), nitric oxide (NO) metabolite, and magnesium (Mg) levels were determined.

**Results:**

The percentage of weight loss and the serum levels of MDA and nitrite in male and female animals were not statistically different. However, the serum levels of BUN, Cr, Mg, and kidney MDA levels, and kidney weight and damage score were significantly greater in males than in females (P < 0.05).

**Conclusions:**

CP-induced nephrotoxicity is gender related for which the mechanisms should be determined.

## 1. Background

Cisplatin (CP) is one of the most common drugs used in clinics to treat different types of cancer such as sarcomas, some carcinomas, germ cell tumors, and lymphomas. However, CP therapy is accompanied by moderate to severe nephrotoxicity. In general, the drug-induced nephrotoxicity may be gender related. The serum levels of creatinine and urea in a model of gentamycin-induced nephrotoxicity were higher in males than females (1, 2). It has been documented that males suffer more from renal injury by Amphotericin B (3) and Tobramycin (4) than females. Moreover, the probability of nephrotoxicity induced by Phenobarbital is higher in male rats than females (5). In addition, male animals are less resistant to ischemic acute renal failure than females (6). It has been reported that the neuropathy induced by CP is gender related (7), and CP interacts with female sex hormones and therefore, estradiol administration prevents the protective role of some antioxidants against CP-induced nephrotoxicity (8). Furthermore, L-arginine and losartan have more protective effects against CP-induced nephrotoxicity in male than in female (9, 10). A preliminary report also indicated that CP-induced nephrotoxicity may be sex related (11). Further studies were suggested to clearly differentiate between CP side effects and the role of gender (7, 11).

## 2. Objectives

The present study was designed to find gender differences of nephrotoxicity induced by daily administration of low doses of CP to rats for two weeks.

## 3. Materials and Methods

Fifteen Wistar rats (male; n = 7, weight 200 ± 6 g; female; n = 8, weight 167 ± 4 g) were randomly divided into two experiment groups. The animals were housed under standard conditions with 12 h light/12 h dark cycles and were given ad libitum access to food and water. The experiments were confirmed to be in accordance with the guidelines of animal ethics committee of Isfahan university of medical sciences.

### 3.1. Experimental Protocols

The animals received CP daily (1 mg/kg/day; ip) for two weeks. CP was purchased from EBEWE PharmaGes.m.b.H (Austria). The body weight of animals was recorded on a daily basis. At the end of the experiment, blood samples were obtained and the rats were then sacrificed. The kidneys were immediately removed and weighed. The left kidney was subjected to histopathological investigations, and the right kidney was homogenized and centrifuged for determination of the supernatant Malondialdehyde (MDA) levels. A sample of liver was also obtained to investigate hepatotoxicity damage induced by CP.

### 3.2. Measurements

Serum creatinine (Cr), blood urea nitrogen (BUN), and magnesium (Mg) levels were determined using quantitative kits (Pars Azmoon, Iran). The serum level of nitrite (stable NO metabolite) was measured using a colorimetric assay kit (Promega Corporation, USA). MDA levels of serum and the supernatant from homogenized tissue were quantified according to the manual methodology (12). Briefly, 500 µL of the sample was mixed with 1000 µL 10% trichloroacetic acid (TCA). The mixture was vigorously shaken and centrifuged at 2000 g for 10 minutes; 500 µL of the supernatant was added to 500 µL 0.67% thiobarbituric acid (TBA). The solution was then incubated in a hot water bath at the temperature of 100 ºC for 10 minutes. After cooling, the absorbance was measured at 532 nm. The concentration of MDA was reported as µmol/L for the serum and as µmol/100 g of tissue for kidney.

### 3.3. Histopathological Procedures

The kidney and liver tissues were fixed in 10% formalin solution, embedded in paraffin for histopathological staining. Hematoxylin and eosin staining was applied to examine the tubular injury. For kidney damage, presence of acute tubular damage such as tubular dilation and simplification, tubular cell swelling and necrosis, tubular casts, and intraluminal cell debris with inflammatory cell infiltration were considered. The intensity of tubular lesions as mentioned above, were scored from 1 to 4, while score zero was assigned to normal tissue without damage. For the liver tissue, any damage including the presence of lymphocytes was determined.

### 3.4. Statistical Analysis

Data are presented as mean ± SEM. To compare the weight change between the groups, repeated measures analysis was applied. The groups were compared with unpaired Student’s t-test for kidney weight, serum levels of BUN, Cr, MDA, Mg, and nitrite; and kidney tissue level of MDA. Mann-Whitney test was employed to compare the pathological damage score among the groups. P-values < 0.05 were considered statistically significant.

## 4. Results

**Figure 1.**
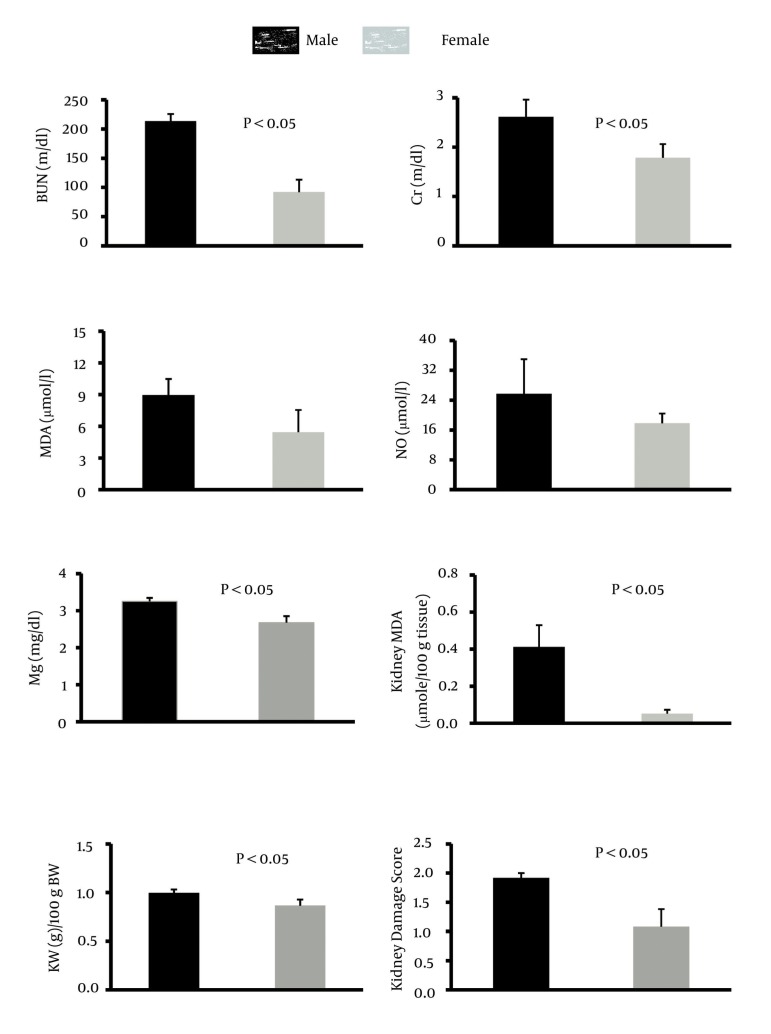
Serum Levels of BUN, Cr, Mg, MDA, and Nitrite, Kidney MDA, Kidney weight/100 g body Weight, and Kidney Damage Scores in Male and Female Animals Treated With CP

**Figure 2.**
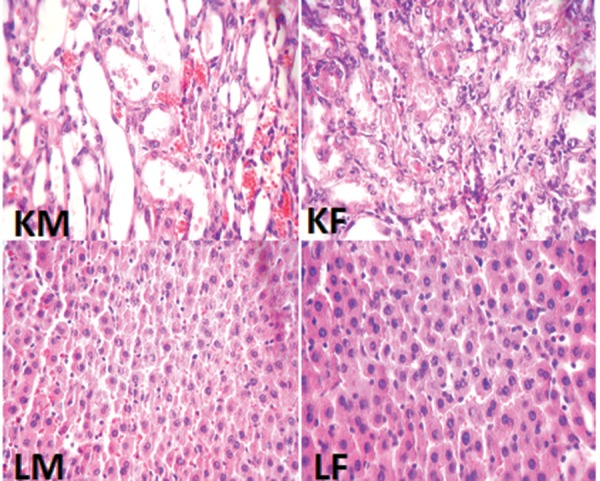
The Images of Kidney and Liver Tissues (magnification x 100). More damage of kidney tissue was observed in male than in female, but no damage was detected in liver tissues of both sexes. KM: kidney tissue of male, KF: kidney tissue of female, LM: liver tissue of male, and LF: liver tissue of female.

The percentage of weight loss in male animals was 13.8 ± 2.1% while female animals lost about 11.8 ± 2.6% of their weight, and the difference between male and female rats was not statistically significant. The data for the serum levels of BUN, Cr, MDA, Mg, and NO, kidney weight and renal MDA level, and pathology damage scores are demonstrated in Figure 1. Except for the serum levels of MDA and NO, the other parameters were significantly different between male and female (P < 0.05) (Figure 1). The pathological data indicated a few lymphocytes in most samples, and no other abnormalities were observed for liver tissue. Therefore, we presume that no damage was induced by CP in the protocol of this study. The images of the kidney and liver tissues of both genders are shown in Figure 2. More damage was observed in the male kidney tissues.

## 5. Discussion

The main purpose of this study was to detect sex-related nephrotoxicity induced by CP. We found that the biochemistry markers of BUN, Cr, Mg, and kidney MDA are significantly different between the two sexes, and the levels of these markers were higher in males. The pathology results also confirmed greater nephrotoxicity intensity in male animals. Some other studies have confirmed the role of sex in drug-induced-nephrotoxicity (3-5, 11). Clinically, hospitalized male patients have been reported to be more vulnerable to nephrotoxicity caused by Amikacin (13). In the presence of CP, male rats excrete more sodium than female rats, which is related to cytotoxicity in kidneys (14). Previously, we presented evidence for sex-based differences in CP-induced nephrotoxicity model and in the current study; we used different doses of CP and treatment durations, which were in agreement with our pervious findings (11). L-arginine and losartan as supplementation in CP-treated animals act differently in male and female animals (9, 10).

The reason for these differences is not clearly known, and at this point it seems that it is not related to female sex hormone, because estrogen itself promotes CP-induced nephrotoxicity (8, 15, 16). Gender difference in renal circulation is another probable reason for sex-related CP-induced nephrotoxicity. Simulation of angiotensin system receptors leads to different responses in the sexes (17, 18) with more vasodilator effect in female, which influences renal blood flow. The renal blood flow on the other hand is disturbed by CP (19). So, probably the female renal blood flow is reduced less by CP than male, which causes less damage.

The affinity of drug binding, pharmacokinetic properties of drugs, and genetic nature are also other factors that may contribute to sex-related different responses (15, 20, 21). These factors are other proposed assumptions for this sex-based difference of lower levels of CP-induced nephrotoxicity in female.

Conclusion: CP-induced nephrotoxicity is gender related due to an unknown mechanism. However, gender differences in renal circulation may be the key factor leading to this difference and this should be verified by further studies.
